# A2-Astrocyte Activation by Short-Term Hypoxia Rescues α-Synuclein Pre-Formed-Fibril-Induced Neuronal Cell Death

**DOI:** 10.3390/biomedicines13030604

**Published:** 2025-03-01

**Authors:** Ha Nyeoung Choi, Seon-Hee Kim, Min Gi Jo, Bina Lee, Young Jin Kim, So Eun Lee, Jeong Hyun Lee, Hye Min Seong, Seong Jae Kim, Sang Won Park, Hye Jung Kim, Heeyoung Kang, Chan Hyun Lee, Min Young Lee, Seung Pil Yun, Minkyeong Kim

**Affiliations:** 1 Department of Pharmacology, Institute of Medical Science, College of Medicine, Gyeongsang National University, Jinju 52727, Republic of Korea; phsl123@naver.com (H.N.C.); seonhkim@gnu.ac.kr (S.-H.K.); iibina@naver.com (B.L.); dog020@naver.com (Y.J.K.); seuu12@naver.com (S.E.L.); uranus-jh@hanmail.net (J.H.L.); parksw@gnu.ac.kr (S.W.P.); hyejungkim@gnu.ac.kr (H.J.K.); 2 Department of Convergence Medical Science, College of Medicine, Gyeongsang National University, Jinju 52727, Republic of Korea; 3 Department of Pathology, College of Medicine, Kyung Hee University, Seoul 02447, Republic of Korea; mingi.jo@khu.ac.kr; 4 Department of Ophthalmology, Institute of Health Sciences, College of Medicine, Gyeongsang National University, Jinju 52727, Republic of Korea; seong_hm@daum.net (H.M.S.); maya12kim@naver.com (S.J.K.); 5 Department of Neurology, Gyeongsang National University Hospital, Jinju 52727, Republic of Korea; miranda75@naver.com (H.K.); princekittyryan@gmail.com (C.H.L.); 6 Department of Neurology, College of Medicine, Gyeongsang National University, Jinju 52727, Republic of Korea; 7 College of Pharmacy, Research Institute of Pharmaceutical Sciences, Vessel-Organ Interaction Research Center (VOICE, MRC), Kyungpook National University, Daegu 41566, Republic of Korea; vetmedic@knu.ac.kr

**Keywords:** Parkinson’s disease (PD), hypoxia, astrocyte, neuronal death, pre-formed fibril

## Abstract

**Background/Objectives:** Parkinson’s disease (PD) is a neuro-degenerative disease for which a radical cure is not available, only symptomatic control. Studies have shown that hypoxia may have disease-modifying effects on PD. **Methods:** Herein, we investigated whether short-term hypoxia activates astrocytes and whether it has a protective effect on pre-formed fibril (PFF)-treated primary cortical neurons. **Results:** Long-term hypoxia suppresses astrocyte activation and induces cell death, whereas short-term hypoxia activates astrocytes without affecting cellular apoptosis or viability. Short-term hypoxia restored the cellular apoptosis and viability of PFF-treated neurons and reduced toxic phospho-α-synuclein (p-α-syn) aggregation. Similarly, the short-term hypoxia-exposed astrocyte-conditioned medium rescued cellular apoptosis and the viability of PFF-treated neurons and p-α-syn expression. Quantitative polymerase chain reaction revealed that short-term hypoxia promotes protective A2 astrocytes and suppresses toxic A1 astrocytes. **Conclusions**: Our findings suggest that short-term hypoxia has a neuro-protective effect against PD by activating protective A2 astrocytes, which rescue PFF-induced neuronal cell death. This provides insights into the clinical implications of short-term hypoxia as a disease-modifying PD strategy.

## 1. Introduction

Parkinson’s disease (PD) is a fast-growing neuro-degenerative disease worldwide, which is characterized by the loss of dopaminergic neurons in the substantia nigra and α-synuclein (α-syn)-laden Lewy bodies [1]. Clinically, patients with PD demonstrate motor symptoms including bradykinesia, rigidity, and resting tremor, which are accompanied by non-motor symptoms, including autonomic dysfunction, cognitive decline, and psychiatric features. Currently, medical treatment with levodopa and deep brain stimulation, which only provide symptomatic control, is available.

Mitochondrial dysfunction and oxidative stress play key roles in the pathogenesis of PD [2]. PD risk genes such as *DJ-1*, *PINK1*, *PRKN*, and *LRRK2* share mitochondrial dysfunction, and 1-methyl-4-phenyl-1,2,3,6-tetrahydropyridine, a complex I inhibitor, induces parkinsonism, which is frequently used in PD mouse models. Based on the link between hypoxia and PD, there is growing evidence that hypoxic conditioning could be a treatment option for PD [3]. Exercise, a well-known protective lifestyle factor in PD, involves exposing the brain to acute intermittent hypoxia, which supports hypoxia-mediated disease-modifying effects [4].

Astrocytes have various physiologic roles, including physical and neurotropic support, maintenance of the blood-brain barrier, regulation of neuro-transmitters and ion concentration, modulation of synaptic transmission, and neural repair [5]. During the adaptive response to hypoxia, molecular pathways involving hypoxia-inducible factor-1α (HIF-1α), NF-kb, and p53 are activated in astrocytes, which induce neuro-protective erythropoietin, vascular endothelial growth factor, and glial-cell-line-derived neurotrophic factor [6].

There are at least two types of astrocytes, toxic A1 and protective A2, and the cellular milieu determines the dominant phenotype [7,8]. In this study, we hypothesized that conditioned hypoxia activates neuro-protective A2 astrocytes.

## 2. Materials and Methods

### 2.1. Animal

All experimental procedures complied with the guidelines outlined in the Laboratory Animal Manual and were approved by the Institutional Animal Care and Use Committee of Gyeongsang National University (Approval No. GNU-210125-M0005). Efforts were made to minimize the number of mice used and to reduce their pain or discomfort throughout this study.

Pregnant ICR mice (13–14 days) were purchased from Koatech Inc. (Ansan-si, Gyeonggi-do, Republic of Korea). Mice were acclimated to a 12 h light/dark cycle and had access to food and water ad libitum. The ambient temperature was maintained at 21 ± 23 °C, and humidity was maintained at 60 ± 10%.

### 2.2. Primary Cortical Neuronal Culture

Whole brains were obtained from DIV 14 pups, the meninges were removed, and primary cortical neurons were isolated and washed three times with DMEM (GenDEPOT, Houston, TX, USA). A single-cell suspension was obtained by trituration. Cell debris and aggregates were removed by passing the single-cell suspension through a 40 μm nylon mesh. The final single-cell suspension was thus achieved and cultured in Neurobasal medium (Gibco, Grand Island, NY, USA) supplemented with B-27 Plus Supplement (Gibco, Grand Island, NY, USA) and penicillin-streptomycin (Gibco, Grand Island, NY, USA) on tissue culture plates coated with poly-D-lysine (Gibco, Grand Island, NY, USA). Half the medium was exchanged every 3–4 days.

### 2.3. Primary Astrocyte Culture

Whole brains from mouse pups at postnatal day 1 were obtained. After removal of the meninges, the brains were washed with DMEM (GenDEPOT, Houston, TX, USA) three times. The brains were transferred to 0.25% Trypsin-EDTA solution followed by 10 min of gentle agitation. DMEM/F12 complete medium was used to stop trypsinization. The brains were washed three times in this medium again. A single-cell suspension was obtained by trituration. Cell debris and aggregates were removed by passing the single-cell suspension through a 100 μm nylon mesh. The final single-cell suspension was thus achieved and cultured in DMEM/F12 (Gibco, Grand Island, NY, USA) supplemented with FBS (Gibco, Grand Island, NY, USA) and penicillin-streptomycin (Gibco, Grand Island, NY, USA) on a T-flask for 13 days. The medium was changed every 3 days. The mixed glial cell population was separated into an astrocyte-rich fraction and a microglia-rich fraction using the EasySep mouse CD11b-positive selection kit (StemCell Technologies Inc., Vancouver, BC, Canada), and the pour-off fraction containing astrocytes was separately cultured.

### 2.4. Preparation of α-Syn PFF

Mouse α-syn monomers (Proteos, Kalamazoo, MI, USA, RP-009) were prepared following the Michael J Fox Foundation’s guidelines. For the generation of PFF, α-syn monomers were agitated in a thermomixer (94× *g* at 37 °C) (ATTO WSC-2630, Tokyo, Japan) for a week, which was followed by sonication for 1 min (20% amplitude) with a 0.5 s pulse on/off cycle 3 times (Sonics & Materials Inc., Newtown, CT, USA). The characterization of PFF is described in our previous study [9].

### 2.5. Preparation of Hypoxia

Hypoxic conditioning was performed in a hypoxic chamber (ASTEC, Fukuoka, Japan) at 37 °C, with 94% N_2_, 5% CO_2_, and 1% O_2_, for 6 and 24 h for long-term exposure, and 30 and 90 min for short-term exposure. Control cells were maintained at 37 °C with 79% N_2_ and 21% O_2_. HIF-1α expression was checked to confirm cellular response to hypoxia.

### 2.6. qPCR Analysis

Total RNA from cultured astrocytes was extracted using the RNeasy Mini Kit (Qiagen, Hilden, Germany) following the manufacturer’s instructions. RNA concentration was measured spectrophotometrically using a Nanodrop spectrophotometer (DeNvix Inc., Dover, DE, USA). Subsequently, 1–2 μg of total RNA was reverse-transcribed to cDNA using the HiSenScript^TM^ RH[−] RT PreMix Kit (Intron Biotechnology, Seongnam-si, Gyeonggi, Republic of Korea). qPCR was performed on the CFX Connect Real-Time PCR System using iQ SYBR Green Supermix (Bio-Rad, Hercules, CA, USA). The expression levels of target genes were normalized to those of GAPDH and calculated based on the comparative cycle threshold Ct method. The names and sequences of primers used in this study are listed in Table S1.

### 2.7. TUNEL Staining/Assay

The apoptosis of neuronal cells was assessed using the In Situ Cell Death Detection Kit (Roche, Basel, Switzerland) and BX53 microscope (Olympus, Tokyo, Japan) according to the manufacturer’s instructions. The TUNEL-positive cell number was divided by the total cell number (DAPI-positive).

### 2.8. Cell Viability Assay

After exposure to hypoxia, cell-cultured media were removed and the cells were incubated with the Cellrix Viability Assay Kit (MediFab, Seoul, Republic of Korea) for 2 h at 37 °C. The VICTOR Nivo Multimode Microplate Reader (PerkinElmer, Waltham, MA, USA) was used to read the absorbance at 450 nm. Background values (measurement of cell culture media only) were subtracted from each well, and the average absorbance values of the triplicates were calculated to indicate cell viability.

### 2.9. AlamarBlue Assay

Cell viability was assessed using an AlamarBlue Cell Viability Reagent (Invitrogen, Waltham, MA, USA). After exposure to hypoxia, cells were incubated with 10% AlamarBlue cell viability reagent for 4 h at 37 °C. The VICTOR Nivo Multimode Microplate Reader (PerkinElmer, Waltham, MA, USA) was used to read the absorbance at 570 nm. Values at 600 nm were used for normalization. The average absorbance of the triplicates was calculated to indicate cell viability.

### 2.10. Immunofluorescence Analysis

Primary cortical neurons were cultured on cover glass in 24-well plates. Primary and secondary antibodies and their dilution are described in Table 1. The amount of positive intensity was measured with ImageJ software (Rasband, W.S., ImageJ, U.S. National Institutes of Health, Bethesda, MD, USA, https://imagej.net/ij/, 1997–2014).

### 2.11. Western Blot Analysis

Primary neurons and astrocytes were harvested and treated by adding protease and a phosphatase inhibitor disposable cocktail (100×) (Thermo Scientific, Waltham, MA, USA) in RIPA buffer (Thermo Fisher Scientific, Waltham, MA, USA). The lysates were centrifuged at 16,022× *g* for 20 min at 4 °C. The supernatant was collected. Samples (20 µg) were mixed with 2× Laemmli buffer (Bio-Rad, Hercules, CA, USA), supplemented with β-mercaptoethanol (Bio-Rad, Hercules, CA, USA), and boiled for 5 min at 95 °C. Proteins were separated by SDS-PAGE and transferred onto the nitrocellulose membranes, 0.45 µm (Bio-Rad, Hercules, CA, USA), for immunoblotting. Immunoblotting was performed with the indicated antibodies shown in Table 1. Immunoblot bands were visualized with chemiluminescence (Pierce, Franklin Park, IL, USA) and photographed using the ChemiDoc image system (Bio-Rad, Hercules, CA, USA). Densitometric analysis of the bands was performed using ImageJ software.

### 2.12. Statistics

Data are presented as the mean ± S.E.M. from at least three biologically independent experiments. All data were analyzed using GraphPad Prism 7.0 software. Differences between the two groups were analyzed with an unpaired *t*-test and differences among multiple groups were analyzed by ANOVA followed by Tukey’s post hoc test. Assessments with *p* < 0.05 were considered significant.

## 3. Results

### 3.1. Long-Term Hypoxia Induced Cell Death of Astrocytes

**Figure 1:**
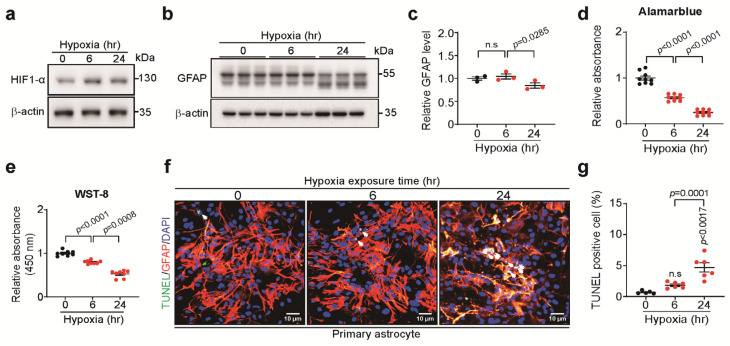
The effect of long-term hypoxia on astrocyte activation and viability. (**a**) Representative Western blot images of HIF-1α in astrocytes under long-term hypoxia. (**b**) Representative Western blot images of GFAP in astrocytes under long-term hypoxia. (**c**) Quantification of GFAP levels shown in (**b**) (*n* = 3). (**d**) Alamar blue assay (*n* = 9). (**e**) WST-8 assay (*n* = 8). (**f**) Representative images of TUNEL (green, arrowhead) staining in astrocytes under long-term hypoxia. DAPI (blue) was used for nuclei staining (scale bar, 10 μm). (**g**) Quantification of (**f**) as the ratio of TUNEL positive to DAPI. Bars represent the mean ± S.E.M. One-way ANOVA followed by Tukey’s post hoc test (*n* = 6). n.s, not significant.

To confirm the effect of long-term hypoxia on astrocytes, primary astrocytes were exposed to 6 and 24 h (h) of hypoxia (37 °C, 94% N_2_, 5% CO_2_, 1% O_2_). Adaptive response to long-term hypoxia was confirmed by HIF-1α expression (Figure 1a). Glial fibrillary acidic protein (GFAP) expression was significantly reduced when hypoxic exposure was prolonged up to 24 h (Figure 1b,c). Astrocyte viability decreased (Figure 1d,e), and cellular apoptosis increased in a time-dependent manner (Figure 1f,g). These results indicate that long-term hypoxia has deleterious effects on astrocytes.

### 3.2. Short-Term Hypoxia Activated Astrocytes Without Cell Death

**Figure 2:**
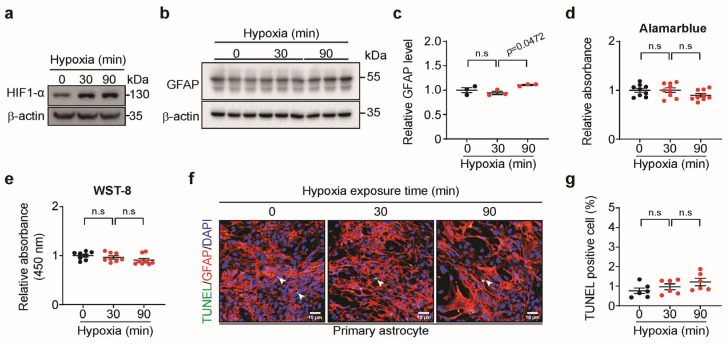
The effect of short-term hypoxia on astrocyte activation and viability. (**a**) Representative Western blot images of HIF-1α in astrocytes under short-term hypoxia. (**b**) Representative Western blot images of GFAP in astrocytes under short-term hypoxia. (**c**) Quantification of GFAP levels shown in (**b**) (*n* = 3). (**d**) AlamarBlue assay (*n* = 9). (**e**) WST-8 assay (*n* = 8). (**f**) Representative images of TUNEL (green, arrowhead) staining in astrocytes under short-term hypoxia. DAPI (blue) was used for nuclei staining (scale bar, 10 μm). (**g**) Quantification of (**f**) as the ratio of TUNEL positive to DAPI. Bars represent the mean ± S.E.M. One-way ANOVA followed by Tukey’s post hoc test (*n* = 6). n.s, not significant.

To confirm the effects of short-term hypoxia on astrocytes, primary astrocytes were exposed to hypoxia for 30 or 90 min (min) of hypoxia. The adaptive response to short-term hypoxia was confirmed by HIF-1α expression (Figure 2a). GFAP expression significantly increased in response to 90 min of hypoxia but was not altered after 30 min of hypoxia (Figure 2b,c). Astrocyte viability (Figure 2d,e) and apoptosis (Figure 2f,g) did not change when the astrocytes were exposed to hypoxia for 30 or 90 min. These results illustrate that 90 min of hypoxia activated astrocytes without causing cell death.

### 3.3. Short-Term Hypoxia Had a Protective Effect on Pre-Formed Fibril (PFF)-Treated Neurons

**Figure 3:**
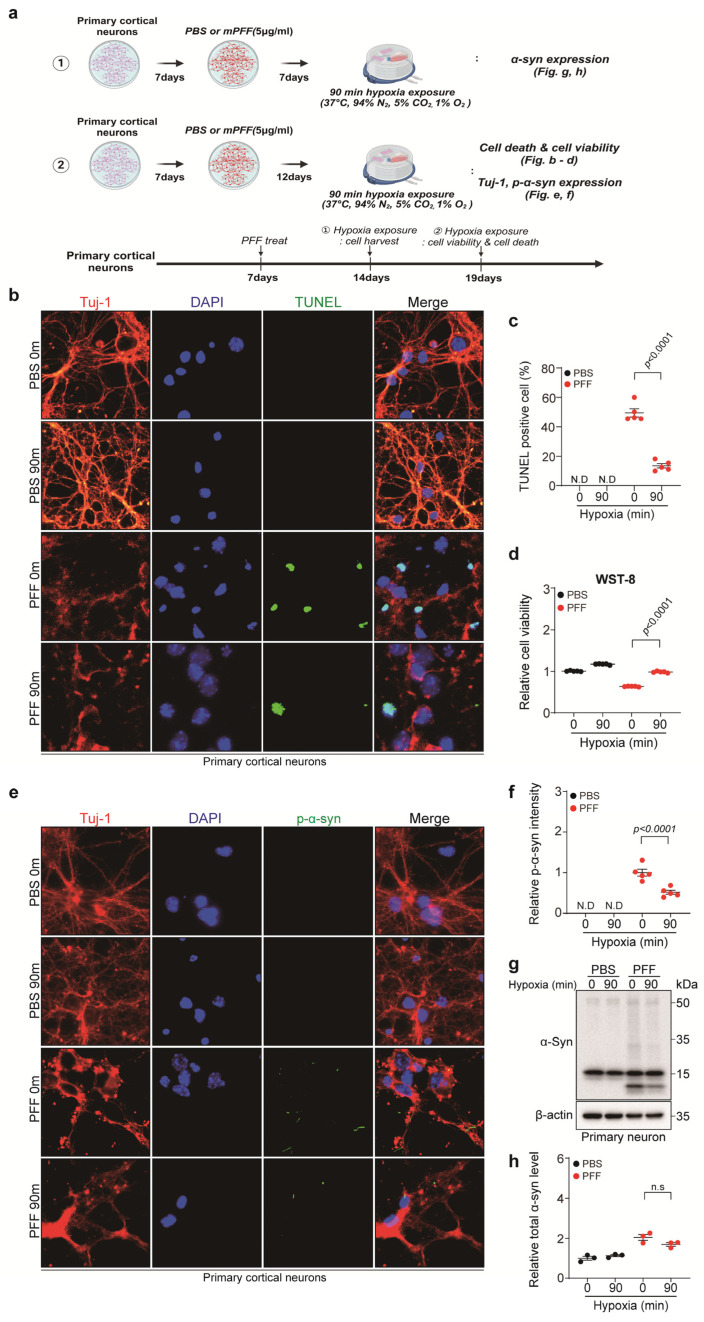
The effect of hypoxia on neurons. (**a**) A schematic summary of the experimental design. (**b**) Representative images of TUNEL (green) and Tuj-1 (red) staining in neurons under 90 min of hypoxia. DAPI (blue) was used for nuclei staining (scale bar, 10 μm). (**c**) Quantification of (**b**) as the ratio of TUNEL positive to DAPI. Bars represent the mean ± S.E.M. An unpaired *t*-test (*n* = 4). (**d**) WST-8 assay (*n* = 5). An unpaired *t*-test. (**e**) Representative images of p-α-syn (green) and Tuj-1 (red) staining in neurons under 90 min of hypoxia. DAPI (blue) was used for nuclei staining (scale bar, 10 μm). (**f**) Quantification of p-α-syn (*n* = 5). Bars represent the mean ± S.E.M. An unpaired *t*-test. (**g**) Representative Western blot images of α-syn in PFF-treated neurons under 90 min of hypoxia. (**h**) Quantification of α-syn levels shown in (**g**) (*n* = 3). Bars represent the mean ± S.E.M. An unpaired *t*-test. N.D, not detected; n.s, not significant.

To confirm the effect of hypoxia on neurons, the primary cortical neurons were treated with PFF 7 days after neuronal culture and exposed to 90 min of hypoxia 7 or 12 days post-PFF treatment (Figure 3a). Hypoxia exposure significantly reduced neuronal apoptosis and increased the viability of PFF-treated neurons and their phosphate-buffered-saline-treated counterparts (Figure 3b–d). Toxic p-α-syn expression was significantly reduced when PFF-treated neurons were exposed to 90 min of hypoxia (Figure 3e,f). While total α-syn expression slightly decreased in PFF-treated neurons in response to 90 min of hypoxia, the difference was not statistically significant (Figure 3g,h). These results suggest that 90 min of hypoxia exerts a protective effect on PFF-treated neurons.

### 3.4. Hypoxia-Exposed Astrocyte-Conditioned Medium (hACM) Rescued PFF-Treated Neuronal Cell Death

**Figure 4:**
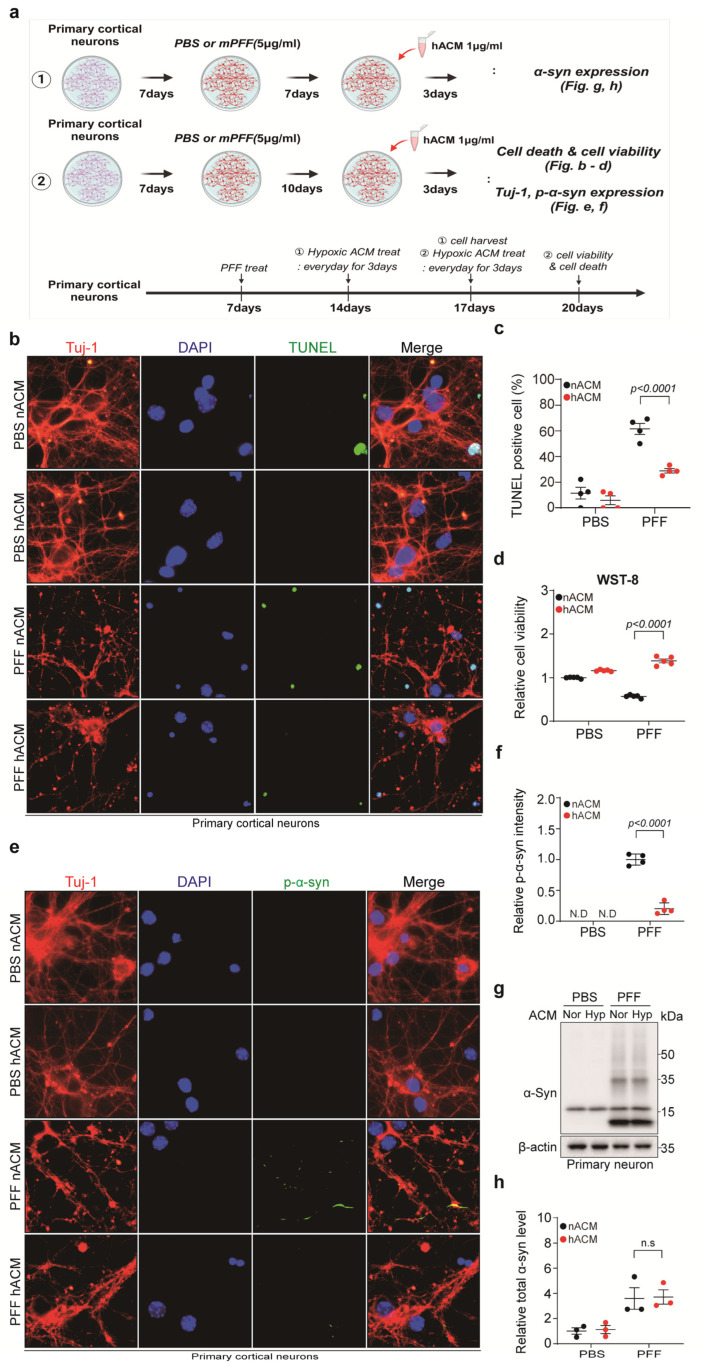
hACM rescued PFF-induced neuronal cell death. (**a**) A schematic summary of the experimental design. (**b**) Representative images of TUNEL (green) and Tuj-1 (red) staining in neurons with hACM administration. DAPI (blue) was used for nuclei staining (scale bar, 10 μm). (**c**) Quantification of TUNEL in (**b**) normalized with DAPI. Bars represent the mean ± S.E.M. An unpaired *t*-test (*n* = 4). (**d**) WST-8 assay (*n* = 5). (**e**) Representative images of p-α-syn (green) and Tuj-1 (red) staining in neurons with hACM administration. DAPI (blue) was used for nuclei staining (dcale bar, 10 μm). (**f**) Quantification of p-α-syn (*n* = 4). Bars represent the mean ± S.E.M. An unpaired *t*-test. (**g**) Representative Western blot images of α-syn in PFF-treated neurons with hACM administration. (**h**) Quantification of α-syn levels shown in (**g**) (*n* = 3). Bars represent the mean ± S.E.M. An unpaired *t*-test. nACM, normoxia-exposed astrocyte-conditioned medium; hACM, 90 min of hypoxia-exposed astrocyte-conditioned medium; N.D, not detected; n.s, not significant.

To confirm the neuro-protective effect of hypoxia-induced activated astrocytes, primary cortical neurons were treated with PFF 7 days after neuronal culture, and 90 min of hACM was administered for 3 days at 7 and 10 days after PFF treatment (Figure 4a). The administration of hACM significantly reduced neuronal apoptosis and increased the viability of PFF-treated neurons (Figure 4b–d). Furthermore, hACM significantly reduced p-α-syn expression in PFF-treated neurons (Figure 4e,f). Total α-syn expression did not significantly differ between PFF-treated neurons with normoxia-exposed ACM and hACM (Figure 4g,h). These results imply that hACM exerts a protective effect on PFF-treated neurons.

### 3.5. Short-Term Hypoxia Provided a Neuro-Protective Effect by Promoting A2 Astrocytes and Suppressing A1 Astrocytes

**Figure 5:**
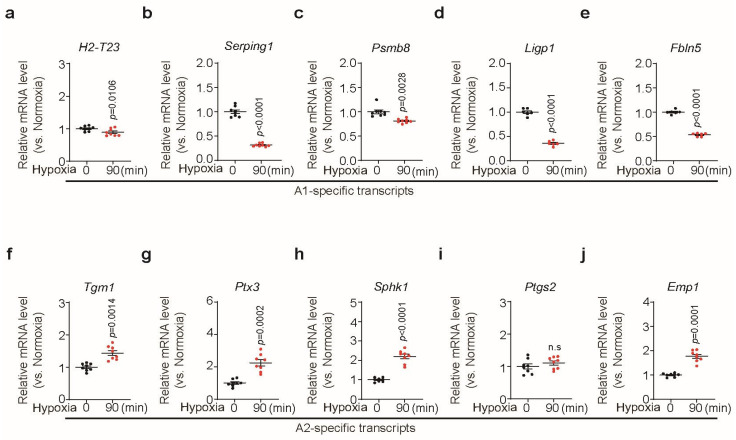
The effect of hypoxia on astrocyte transcripts. (**a**–**e**) Quantitative polymerase chain reaction (qPCR) analysis of A1-specific transcripts in response to 90 min of hypoxia (*n* = 8). (**f**–**j**) qPCR analysis of A2-specific transcripts in response to 90 min of hypoxia (*n* = 8). Bars represent the mean ± S.E.M. An unpaired *t*-test. n.s, not significant.

When astrocytes were exposed to 90 min of hypoxia, A1-specific mRNA levels significantly decreased (Figure 5a–e), whereas A2-specific mRNA levels significantly increased (Figure 5f–j). These results indicate that protective A2 astrocytes were activated, whereas toxic A1 astrocytes were suppressed in response to short-term hypoxia. The raw data of qPCR are separately described in Table S2.

## 4. Discussion

The major finding of this study was that short-term hACM treatment rescued PFF-induced neuronal death. Mechanistically, short-term hypoxia reduces toxic p-α-syn expression by activating protective A2 astrocytes.

Hypoxia can trigger neuro-degeneration or, conversely, provide neuro-protection by regulating inflammation, oxidative stress, and mitochondrial function depending on the severity, duration, and frequency [10]. We first investigated the appropriate hypoxic conditions in which astrocytes are activated without damage. The oxygen level of the brain in the physiologic condition is far lower than that of the ambient air and varies among organs, even within the brain structures (e.g., cortex and brainstem), ranging from 1% to 5 [11]. As we used primary whole-brain astrocytes and cortical neurons, 1% O_2_ was settled for hypoxia. Astrocytes were exposed to both long- and short-term hypoxia to determine the duration of exposure. When long-term hypoxia was induced, astrocyte activation and viability decreased and cellular apoptosis increased with prolonged exposure time. However, 90 min of short-term hypoxia activated astrocytes without affecting their viability or inducing apoptosis. These results are in line with previous studies that showed that astrocyte viability decreased in a time-dependent manner under hypoxic conditions [7,12]. As neurons are more susceptible to hypoxic insult than astrocytes, we also confirmed that 90 min of hypoxia decreased neuronal apoptosis and increased cell viability (Figure 3b–d) [13]. As a result, 90 min of exposure time was settled for our research.

This study was motivated by the notion that conditioned hypoxia may exhibit a disease-modifying effect in PD [3,14]. To simulate the intervention of PD progression, PFF-treated neurons were exposed to 90 min of hypoxia at 12 days post-PFF treatment, where toxic p-α-syn aggregates appear in neuronal axons, cell bodies, and dendrites albeit without neuronal death [15]. We found that cellular apoptosis was reduced, and cell viability was enhanced in PFF-treated neurons when exposed to 90 min of hypoxia. Interestingly, p-α-syn expression levels were significantly reduced when 90 min of hypoxia was administered. Instead of directly exposing neurons to hypoxia, hACM was added to PFF-treated neurons for 3 days, at 10 days post-PFF treatment. Similar to the direct effects of hypoxia, administering hACM rescued neuronal apoptosis and improved cell viability. Furthermore, p-α-syn expression levels were significantly reduced when hACM was administered. Our results imply that short-term hypoxia exerts a protective effect on PFF-treated primary cortical neurons, which is, at least in part, mediated by astrocytes. Under conditioned hypoxia, activated astrocytes seem to ameliorate p-α-syn aggregation, a critical process for PD pathology and progression [16].

Our quantitative polymerase chain reaction (qPCR) results account for the neuro-protective function of activated astrocytes under short-term hypoxia. When astrocytes were exposed to 90 min of hypoxia, the expression of H2-T23, Serping1, Psmb8, Ligp1, and Fbln5 was significantly reduced, whereas that of Tgm1, Ptx3, Sphk1, and Emp1 was significantly enhanced. H2-T23, Serping1, Psmb8, Ligp1, and Fbln5 belong to A1 astrocyte markers that participate in antigen presentation; regulate the complement pathway, interferon signaling, or protein ubiquitination; or interact with the extracellular matrix in response to inflammation. Conversely, Tgm1, Ptx3, Sphk1, and Emp1 are A2 astrocyte markers that encode proteins involved in immune response regulation, signal transduction, and cell cycle control. These proteins show increased expression following ischemic insult in vivo, consistent with our findings [7]. Recent studies suggest that astrocyte dysfunction contributes to PD pathogenesis, and modulating astrocyte phenotypes may help mitigate disease progression [17,18]. Specifically, microglia activate A1 astrocytes through IL-1α, TNF, and C1q, leading to complement cascade upregulation and the secretion of neurotoxins that induce rapid neuronal death [19]. In contrast, A2 astrocytes promote neuronal recovery by upregulating neurotrophic factors and cytokines such as CLCF1, LIF, IL6, and thrombospondins, which support neuronal function [7,19]. Moreover, A2 astrocytes induce the neuroprotective nuclear factor erythroid 2-related factor (Nrf2) and enhance glutamate uptake, thereby preventing excitotoxicity [20]. Our results suggest that conditioned hypoxia holds therapeutic potential for PD by promoting A2 astrocytes and suppressing A1 astrocytes.

Mitochondrial dysfunction and oxidative stress are pivotal contributors to PD pathogenesis [21]. Dopaminergic neurons in the substantia nigra are particularly vulnerable to oxidative stress, and the antioxidant deficit observed in PD may exacerbate reactive oxygen species accumulation, ultimately leading to neuronal cell death [22,23]. Adaptive responses to hypoxia, including the activation of HIF and Nrf2, mitigate oxidative stress, enhance α-synuclein clearance, and reduce nigrostriatal neurodegeneration [24,25]. These mechanisms have been targeted in several therapeutic interventions for PD [14]. Additionally, although it is unclear whether hACM ceased p-α-syn accumulation, reversed the toxic aggregates, or both, based on our study, hACM or short-term hypoxia potentially exhibits a disease-modifying effect for PD as 10 days of post-PFF treatment marks the initiation of p-α-syn-induced neuronal dysfunction. Considering that the first PD motor symptoms appear when more than 50% of the dopaminergic neurons are lost [26], it is crucial to investigate whether hACM can halt PD progression in future studies.

The most critical limitation of our study is the lack of in vivo data. Nevertheless, we used primary cells with well-validated genomic and phenotypic stability. In the future, we aim to extend our research to PD mouse models or human-iPS-cell-derived dopaminergic neurons to further validate our findings. In addition, while astrocytes are known to present morphological changes in response to external stimuli such as hypoxia, our study did not observe the expected changes. This discrepancy may be due to differences in experimental protocol, such as the hypoxic exposure setup and cell culture conditions. To address this limitation, future studies should investigate the morphological changes of astrocytes in response to both long- and short-term hypoxia exposure and correlate these findings with changes in astrocytic phenotype. Finally, although we only searched for astrocyte transcripts, other mechanisms, such as inhibiting pro-inflammatory cytokines or oxidative stress, are also possible [27,28]. As our qPCR data indicate, we believe the immune response is deeply involved in the adaptive response to hypoxia. Accordingly, microglia, an upstream marker of astrocyte activation, or inflammatory markers could also be investigated for further mechanistic analyses [29].

**Figure 6:**
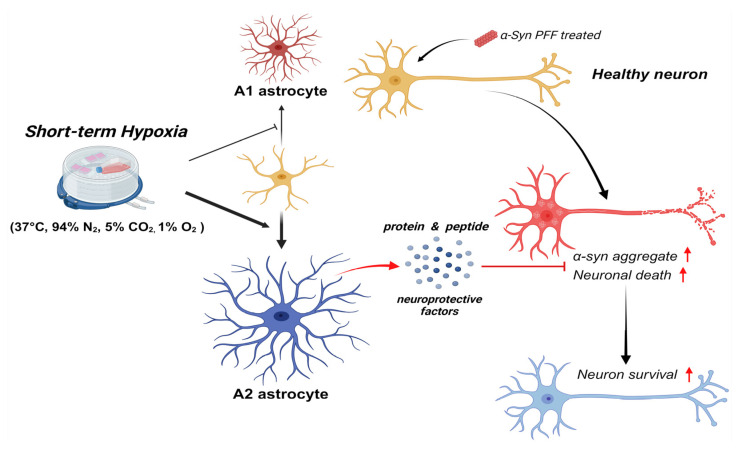
A schematic showing that short-term hypoxia exerts neuroprotective effects in PD by activating protective A2 astrocytes and increasing the survival of a-syn PFF-treated neurons through the expression of neuroprotective factors.

In conclusion, our study suggests that short-term hypoxia exerts neuro-protective effects in PD by activating protective A2 astrocytes (Figure 6). This study provides insights into the clinical implications of short-term hypoxia as a disease-modifying strategy for PD.

## Figures and Tables

**Table 1:** List of antibodies used in the present study.

Antibodies	Company	Catalog No.	Concentration
p-α-synuclein (Ser129)	Biolegend (San Diego, CA, USA)	825701	IF 1:1000
α-synuclein	BD Bioscience (Herlev, Denmark)	610787	WB 1:1000
β-actin-HRP	Sigma-Aldrich (St. Louis, MO, USA)	A3854	WB 1:20,000
GFAP	EMD Millipore (Burlington, MA, USA) Dako (Santa Clara, CA, USA)	MAB360 Z033429	WB 1:1000 IF 1:500
Tuj-1	Biolegend (San Diego, CA, USA)	802001	IF 1:1000
HIF-1α	Cell Signaling (St. Louis, MO, USA)	14179	WB 1:500
Cy^TM^3 AffiniPure Donkey Anti-Rabbit IgG (H + L)	Jackson (West Grove, PA, USA)	711-165-152	IF 1:500
Fluorescein (FITC) AffiniPure Donkey Anti-Mouse IgG (H + L)	Jackson	715-095-151	IF 1:500
Goat anti-Mouse IgG-heavy- and light-chain Antibody HRP Conjugated	BETHYL (Montgomery, TX, USA)	A90-116P	WB 1:10,000
Peroxidase-AffiniPure Goat Anti-Rabbit IgG (H + L)	Jackson	111-035-144	WB 1:10,000

No., number; IF, immunofluorescence; WB, Western blot; HRP, horseradish peroxidase; GFAP, glial fibrillary acidic protein; HIF, hypoxia-inducible factor.

## Data Availability

Correspondence and requests for materials and access to datasets should be addressed to S.P.Y. or M.K. All study data are included in this article.

## Supplementary Materials

The following supporting information can be downloaded at https://www.mdpi.com/article/10.3390/biomedicines13030604/s1. Figure S1: Original full blots images of Western blot; Table S1: List of the primers used for quantitative polymerase chain reaction analyses; Table S2: Raw data of qPCR (A1 and A2).
