# Supplementary material for: A2-Astrocyte Activation by Short-Term Hypoxia Rescues α-Synuclein Pre-Formed-Fibril-Induced Neuronal Cell Death
Source: Biomedicines. 2025 Mar 1;13(3):604. doi: 10.3390/biomedicines13030604 (PMC11940376; doi:10.3390/biomedicines13030604)

Figure S1. Original full blot images

Figure 1a
HIF1-α

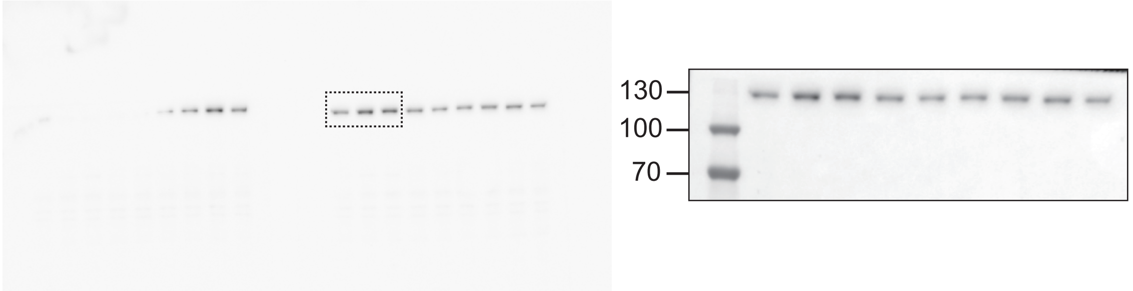

Figure 1a
β-actin

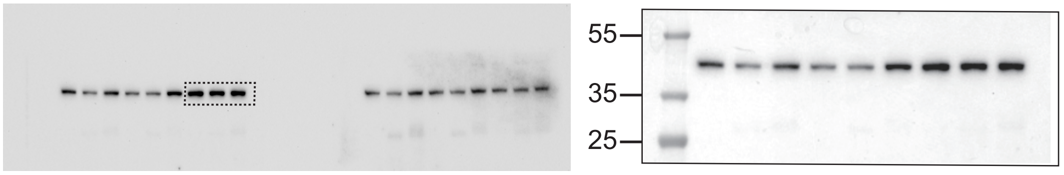

Figure 1b
GFAP

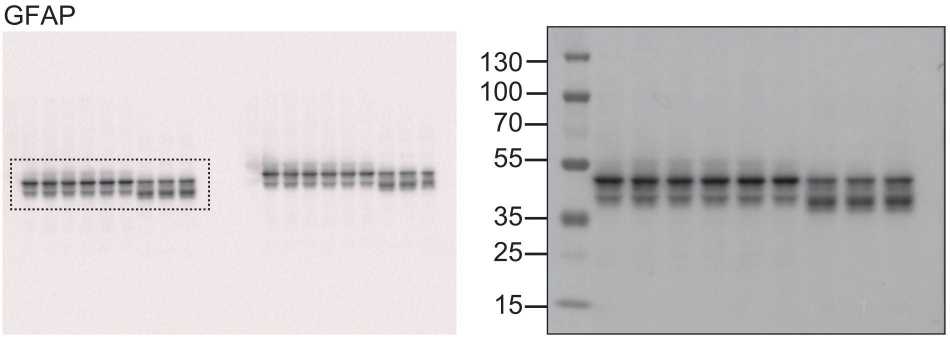

Figure 1b
β-actin

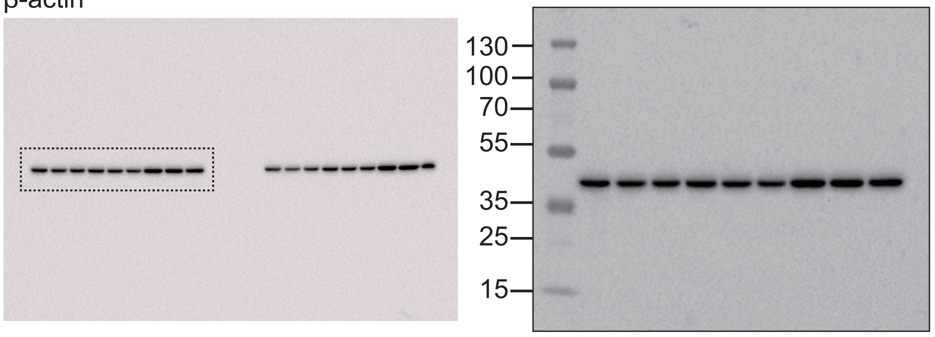

Figure 2a
HIF1-α

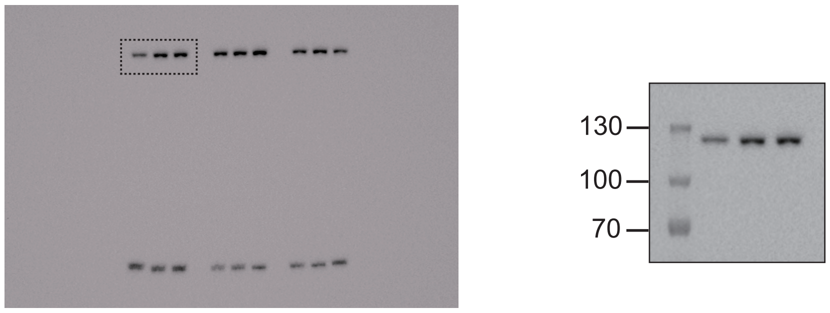

Figure 2a
β-actin

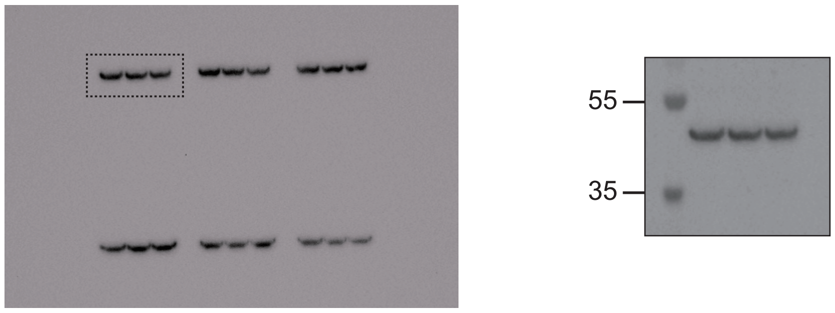

Figure 2b
GFAP

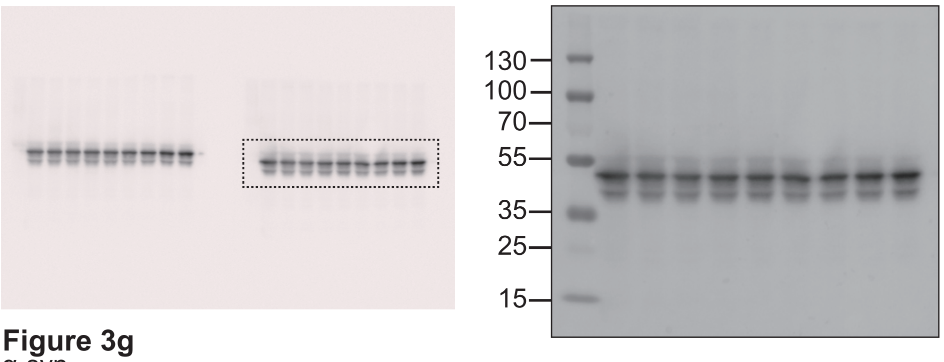

Figure 2b
β-actin

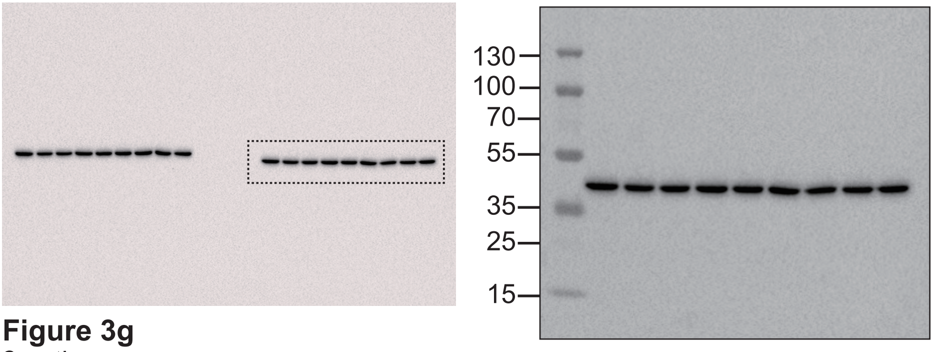

Figure 3g
α-syn

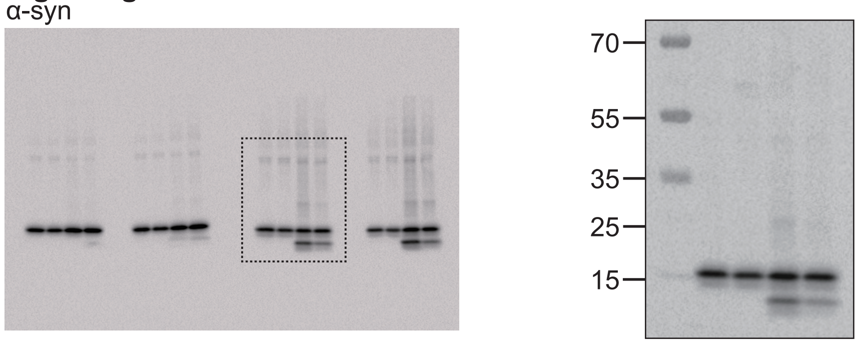

Figure 3g
β-actin

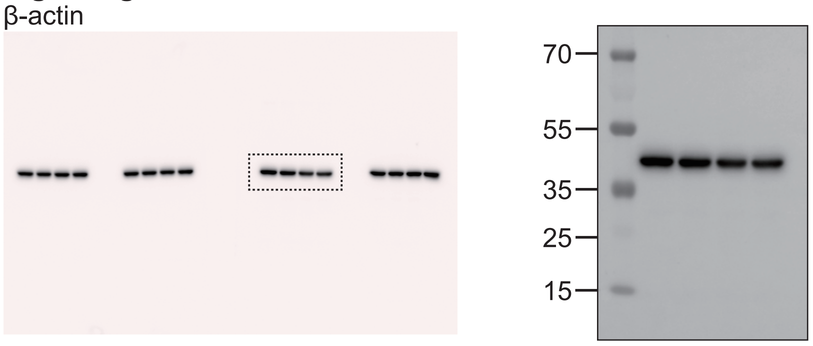

Figure 4g
α-syn

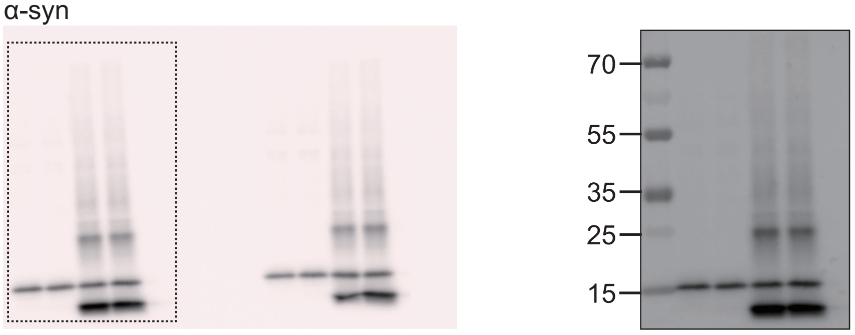

Figure 4g
β-actin

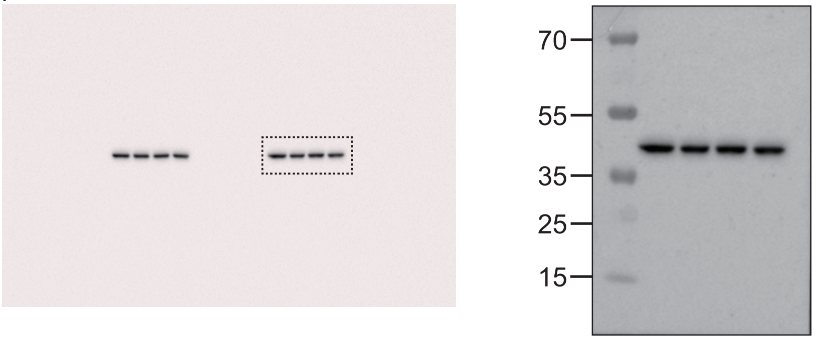

Supplement: Supplementary file 1 [file biomedicines-13-00604-s001.zip › Figure S1-Original full blots .pdf]
