# Supplementary material for: A2-Astrocyte Activation by Short-Term Hypoxia Rescues α-Synuclein Pre-Formed-Fibril-Induced Neuronal Cell Death
Source: Biomedicines. 2025 Mar 1;13(3):604. doi: 10.3390/biomedicines13030604 (PMC11940376; doi:10.3390/biomedicines13030604)
Supplement: Supplementary file 1 [file biomedicines-13-00604-s001.zip › Supplement Table__Biomedicines .pdf]

Supplementary Materials

Table S1 List of the primers used for quantitative polymerase chain reaction analyses

	Gene	Forward primer	Reverse primer
**A1**	H2-T23	GGACCGCGAATGACATAGC	GCACCTCAGGGTGACTTCAT
	Serping1	ACAGCCCCCTCTGAATTCTT	GGATGCTCTCCAAGTTGCTC
	Psmb8	CAGTCCTGAAGAGGCCTACG	CACTTTCACCCAACCGTCTT
	Ligp1	GGGGCAATAGCTCATTGGTA	ACCTCGAAGACATCCCCTTT
	Fbln5	CTTCAGATGCAAGCAACAA	AGGCAGTGTCAGAGGCCTTA
**A2**	Tgm1	CTGTTGGTCCCGTCCCAAA	GGACCTTCCATTGTGCCTGG
	Ptx3	AACAAGCTCTGTTGCCCATT	TCCCAAATGGAACATTGGAT
	Sphk1	GATGCATGAGGTGGTGAATG	TGCTCGTACCCAGCATAGTG
	Ptgs2	GCTGTACAAGCAGTGGCAAA	CCCCAAAGATAGCATCTGGA
	Emp1	GAGACACTGGCCAGAAAAGC	TAAAAGGCAAGGGAATGCAC
	GAPDH	TGGCAAAGTGGAGATTGTTG	TTGACTGTGCCGTTGAATTTG

Table S2 Raw data of qPCR (A1 and A2)

	mRNA	Normoxia	Hypoxia (90min)	t-test
A1	H2-T23	1	0.11 ± 0.05	< 0.05
	Serping1	1	0.69 ± 0.04	< 0.0001
	Ligp1	1	0.64 ± 0.04	< 0.0001
	Fbln5	1	0.47 ± 0.02	< 0.0001
	Psmb8	1	0.19 ± 0.04	< 0.001
A2	Tgm1	1	0.44 ± 0.09	< 0.001
	Ptx3	1	1.24 ± 0.20	< 0.0001
	Sphk1	1	1.19 ± 0.12	< 0.0001
	Ptgs2	1	0.11 ± 0.10	n.s
	Emp1	1	0.77 ± 0.09	< 0.0001

The values represent the mean ± S.E.M (n = 8). An unpaired t-test was used for statis tical analyses.
